# En bloc resection of pararectal ectopic thyroid tissue: a case report

**DOI:** 10.1093/jscr/rjad515

**Published:** 2023-09-30

**Authors:** Janyssa Charbonneau, Véronique Lefebvre, Philippe Bouchard

**Affiliations:** Surgery Department, Laval University, Quebec City, Quebec, Canada; Anatomopathology Department, Laval University, Quebec City, Quebec, Canada; Surgery Department, Laval University, Quebec City, Quebec, Canada

**Keywords:** ectopic thyroid tissue, ectopic thyroid gland, ectopic thyroid adenoma, pararectal mass, pararectal tumor, case report

## Abstract

A pararectal mass’ specific diagnosis can be challenging as a broad range of both benign and malignant tumors are possible. Many of these lesions are congenital and do not require treatment, if asymptomatic. Special attention is to be paid when imaging findings are not typical. In such cases, definitive diagnostic can require surgical excision. To this day, ectopic thyroid tissue was not part of known differential diagnosis. This is the first reported case of thyroid adenoma found in the perirectal area. Ectopic thyroid gland can progress over time and include malignant transformation, although rare. It needs to be considered when managing these cases, especially in unusual locations. This case report offers a systematic approach to the atypical pararectal tumor. It shares new specific clinical experience in managing a case  of pararectal ectopic thyroid adenoma, from both a surgical and a histopathological point of view.

## Introduction

Differential diagnosis for pararectal tumors is wide, but predominantly benign. Many congenital lesions need to be considered, such as dermoid cysts, tailgut cysts, teratomas, or lymphangiomas. Although rare, neurofibromas and perineal schwannomas can also be evoked. Submucosal or exophytic rectal tumors are among possible diagnoses, including gastrointestinal stromal tumors (GIST), neuroendocrine tumors (NET), leiomyomas, leiomyosarcomas, and hemangiomas [1]. Endometriomas, abscesses, and duplication cysts are also considered [2]. Diagnosis is obtained with a combination of imaging studies, with or without tissue samples. In some cases, surgical excision will be required for definitive diagnosis.

## Case report

### Patient information

**Figure 1:**
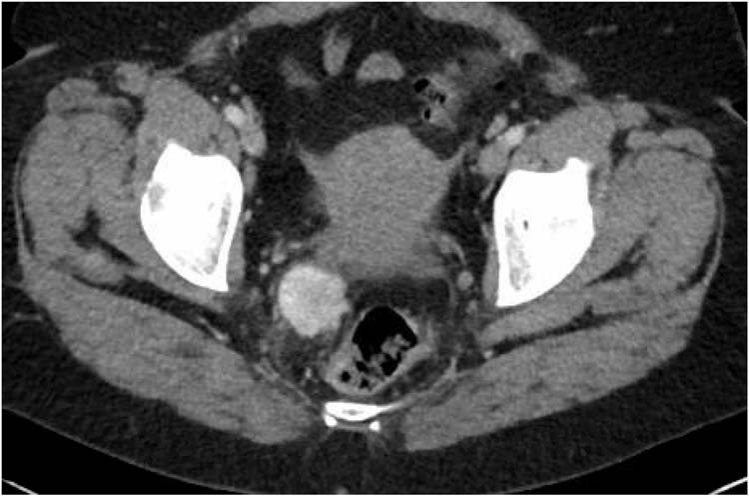
Latest CT evaluation prior to consultation, showing a right pararectal hypervascular mass.

This is the case of a healthy 66-year-old female, referred to a colorectal surgery center for a slow-progressing pararectal mass of 14 years. This incidental finding had since been followed by computed tomography (CT). The mass had progressed from 19 mm to 34 mm (Fig. 1). She had a single dysplastic rectal polyp resected 2 years earlier, but no history of malignancy. She was asymptomatic, apart from a recently growing pain in her right groin.

### Diagnostic assessment

**Figure 2:**
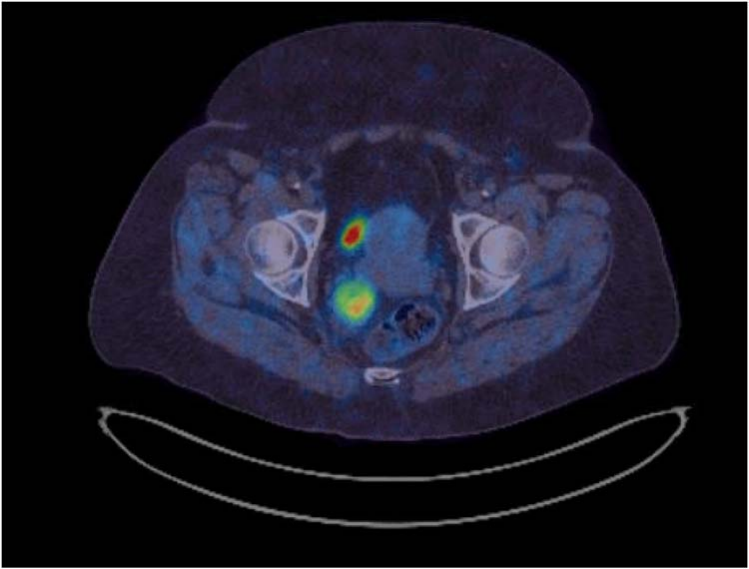
Hypersignal on Gallium-68 Dotatate PET scan (SUVmax 9.4).

**Figure 3:**
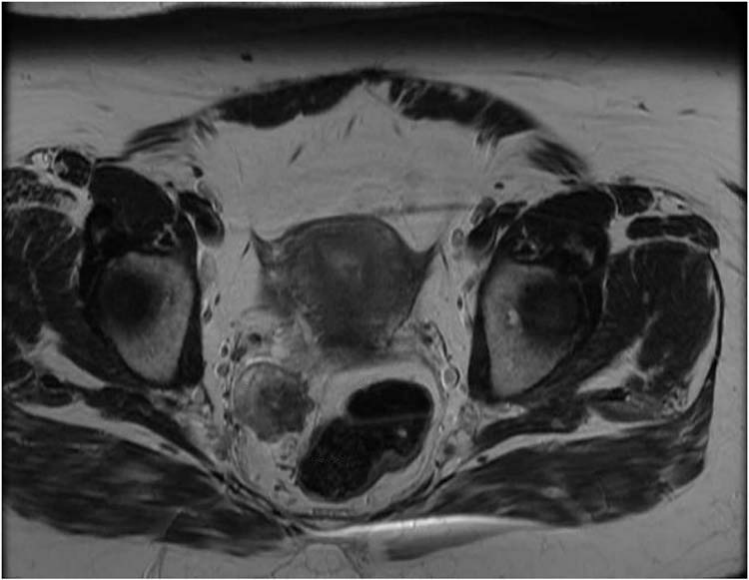
Latest pelvic MRI on initial consultation (T2-weighted images).

Her physical exam was normal. A right extraluminal mass was barely palpable on digital rectal examination. The pararectal mass showed no hypermetabolic activity on recent positron emission tomography (PET) scan. Either a GIST or a NET was suspected. Gallium-68 Dotatate PET scan revealed high expression of somatostatin receptors (Fig. 2). Pelvic magnetic resonance imaging (MRI) showed an isodense lesion, slightly heterogenous, on T1-weighted images with persistent T2 hyperintensity and restricted diffusion (Fig. 3). It was infiltrating the right lateral mesorectum without direct contact with the rectal wall. Colonoscopy showed no endoluminal lesion. Two ultrasound-guided fine needle aspirations (EUS-FNA) were unconclusive and patient developed osteodiscitis. Transgluteal CT-guided biopsy finally revealed the presence of thyroid tissue. Listed diagnoses were teratoma, tailgut cyst, or less likely, thyroid carcinoma. Subsequent thyroid ultrasonography revealed four non-specific micronodules. Thyroid function tests were normal, except for primary hyperparathyroidism. Parathyroid sestamibi scan suggested a single adenoma. A multidisciplinary team was made up of colorectal, head and neck, and orthopedic surgeons, along with an endocrinologist. The case was discussed at the Colorectal Tumor Board. A collaborative two-step surgical approach was proposed.

### Surgery

**Figure 4:**
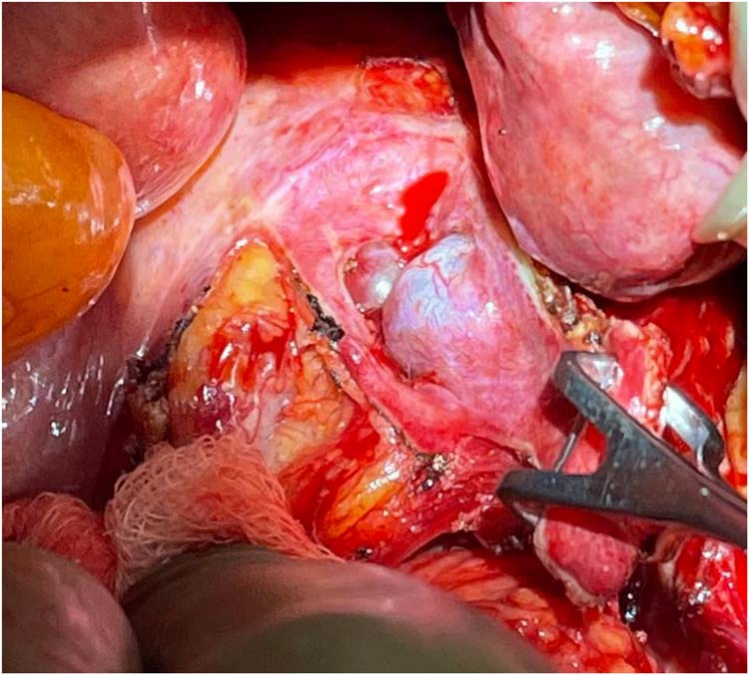
Perioperative location of the tumor, as the rectum is being retracted to the left (by hand).

**Figure 5:**
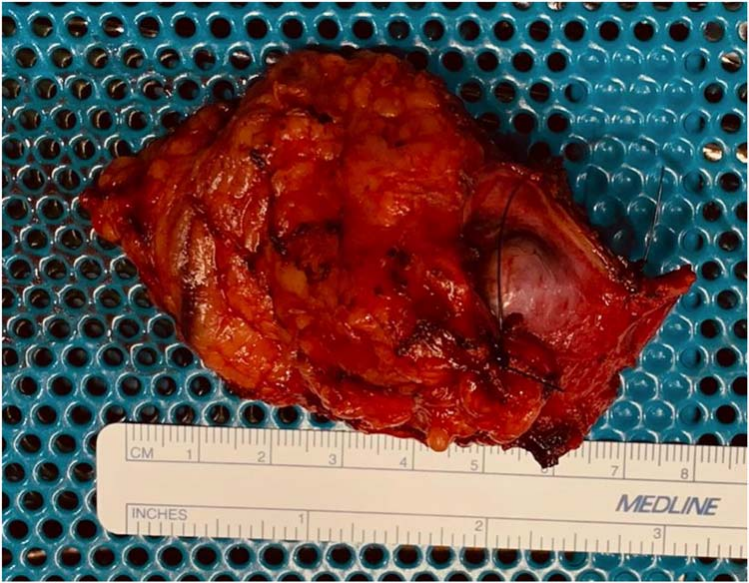
Macroscopic examination of the excised pararectal mass.

Patient underwent laparotomy with *en bloc* resection of pararectal mass with right lateral mesorectum, right Fallopian tube and ovary (Fig. 4). A complete iliac lymphadenectomy was executed as multiple necrotic lymph nodes were found along the common iliac vessels. The mass was removed with negative macroscopic margins, free from the rectum wall (Fig. 5). Presacral drainage and disk debridement was completed for refractory osteodiscitis. Four months later, patient underwent total thyroidectomy with selective parathyroidectomy.

### Pathology

Routine histology was performed with hematoxylin and eosin stain. Immunohistochemistry testing used BerEP4, Calretinin, CD10, MOC31, PAX8, TTF1, and WT1 antibodies. Benign-appearing thyroid tissue was found. Margins were negative. All six lymph nodes were negative. Specific diagnosis remained uncleared, but metastasis from thyroid follicular carcinoma could not be entirely excluded. Specimens were sent to University of Pittsburgh Medical Center (UPMC)Presbyterian’s specialized pathology laboratory in Pittsburgh, where additional molecular studies were completed. Final diagnosis was a 3.6 cm thyroid follicular adenoma, with capsular irregularities insufficient for proper invasion. *ThyroSeq* testing, a set of molecular analysis for thyroid nodules to identify genomic alterations, was performed. Two classes of alterations were identified: chromosomal abnormalities occasionally found in teratomas, consistent with the tumor’s clonal nature, and non-splice site EIF1AX mutation, typically found in follicular adenomas, with a 5% low-risk cancer association. Thyroidectomy’s pathology findings included minimal nodular thyroid hyperplasia with a 0.4 cm nodule. Follicular cells appeared slightly different from those in the pararectal mass. Papillary thyroid microcarcinoma (pT1a, <0.1 cm) was also revealed. Parathyroid demonstrated hyperplasia.

### Follow-up and prognosis

Six months postoperative, thyroid scintigraphy demonstrated residual functional tissue at thyroidectomy’s site, but no other iodine uptake. Ultimately, this was a case of benign adenoma transformation of ectopic thyroid tissue (ETT). With complete excision and the absence of malignity, no long-term colorectal follow-up was deemed necessary. No radioiodine therapy was indicated. Survival is not compromised. Risk of recurrence remains unclear as it is rarely discussed in limited existing literature [3].

## Discussion

There is no reported case of pararectal metastasis from thyroid cancer. Pelvic thyroid tissue, even benign, is an unusual finding. ETT overall prevalence is 1 per 100 000–400 000 [4, 5]. Most reported cases have been found in the head and neck region [5]. One case of thyroid microfollicular adenoma of the lung has been recorded [6]. Subdiaphragmatic locations are especially unusual, considering thyroid’s normal embryological descent stops at the trachea. Very rare cases of ETT have been described in small intestine’s mesentery [7], pancreas [8], gallbladder [9], adrenal gland [10], or around porta hepatis [11]. A few more gynecological ETTs, including struma ovarii, have been recorded [4]. ETT diagnosis is more frequently made in females, under 30 years old. Puberty and pregnancy can expedite diagnosis because of increased hormonal demands triggering ectopic glands growth. No specific gene mutations have been associated yet. Most patients remain asymptomatic, but induced hypothyroidism, hyperthyroidism, or compressive symptoms can occur. Suspected ETT can be evaluated by ultrasounds, CT scan, or MRI. Technetium-99, Iodine-131, or Iodine-123 scintigraphy can identify functional ectopic tissue. FNA usually confirms diagnosis and enables the ruling out of non-thyroid malignancies [4]. In definitive ETT cases, asymptomatic euthyroid patients do not require treatment. Follow-up is still indicated to ensure no malignancy develops, although rare [12, 13]. In the case herein presented, patient was symptomatic as her lesion progressed. A thyroid cancer metastasis was feared; therefore, surgery was mandatory. Surgical removal is otherwise indicated for patients with symptoms or hormonal imbalances. If orthotopic thyroid appears normal on ultrasound, ectopic tissue can typically be removed without risking hypothyroidism [4]. Hormonal suppressive therapy should be favored according to some authors [14]. Others state that surgery should prevailed, when possible, because of potential malignant transformation [5]. Radioiodine therapy is usually limited to patients who are not surgical candidates [4].

This is the first reported case of pararectal ETT. More common diagnoses need to be excluded first-hand, but ETT can now be evoked. In such rare, suspected cases, surgical resection should prevail to ensure right diagnosis, especially in progressing or symptomatic lesions.

## Data Availability

This patient's non-nominal data is available from corresponding author (JC), upon reasonable request.
